# *Bacillus Methylotrophicus* Has Potential Applications Against *Monilinia Fructicola*

**DOI:** 10.1515/biol-2019-0046

**Published:** 2019-11-06

**Authors:** Xue Yuan, Xu Hou, Haotian Chang, Rui Yang, Fang Wang, Yueping Liu

**Affiliations:** 1 Key Laboratory for Northern Urban Agriculture Ministry of Agriculture and Rural Affairs, College of Bioscience and Resources Environment, Beijing University of Agriculture, Beijing 102206, China; 2 Food Science and Engineering College, Beijing University of Agriculture, Beijing 102206, China; 3 Beijing Key Laboratory of New Technique in Agricultural Application, Beijing University of Agriculture, Beijing 102206, China

**Keywords:** *B. methylotrophicus* strain XJ-C, peaches, *M. fructicola*, morphological effects, biocontrol

## Abstract

Biocontrol is a cost-effective and environmentally friendly technique used in agricultural production. We isolated and screened a bacterial strain from the soils of a peach orchard with high yield. Using biochemical and physiological analysis as well as phylogenetic sequencing data, we identified a strain of *Bacillus methylotrophicus*, strain XJ-C. The results of our screening trials showed that XJ-C was able to suppress *M. fructicola* at an inhibition rate of 81.57%. Following the application of a 1×10^9^ CFU/mL XJ-C strain suspension to the fruits, leaves, and shoots of peach trees infected with *M. fructicola*, the inhibition rate reached 64.31%, 97.34%, and 64.28%, respectively. Using OM and SEM, we observed that, under the inhibition of strain XJ-C, *M. fructicola* mycelium and spores were abnormally shaped. Under TEM, cell walls were transparent, organelles had disappeared, and the intracellular vacuole was deformed. Thus, XJ-C has the potential to be used in biocontrol.

## 1 Introduction

During tree development, the fruits, leaves, and shoots of peach trees are at increased risk of infection by *M. fructicola*, also known as sclerotinia [1, 2]. Particularly in the late stages of fruit development, *M. fructicola* can cause peach fruits to rot, affecting even 80% to 90% of fruits in certain environmental conditions such as high temperatures and wet weather [3]. This results in major economic losses due to inferior fruit quality. Current agricultural practices utilize chemical fungicides to prevent and control *M. fructicola* [4]. However, due to the emergence of resistant pathogenic strains, chemical fungicide application may not avert commercial failure [5, 6, 7]. As people become more aware of food safety, the drawbacks of chemical pesticides, such as toxic residues, environmental pollution, etc., have become increasingly obvious [8, 9, 10]. Therefore, biological control using beneficial microbes can provide an alternative and complementary strategy, owing to its minimal toxic residues, lower pollution potential, and similar and potentially even better efficacy as compared to chemical control [11, 12, 13]. For example, *Trichoderma harzianum* AS12-2 strain significantly controlled rice sheath blight better even than propiconazole, the most commonly used fungicide in Iran [14]. The antagonistic radii (mm) of carbendazim and *Bacillus subtilis* against *Fusarium oxysporum* were 9.6 and 7.5, respectively, and the effectiveness of biocontrol and chemical control were comparable in the study [15].

Previous research has shown that certain *Bacillus* spp. can effectively inhibit diseases of fruits and vegetables. *B. cereus* produces a vast array of biologically active molecules that work effectively against *Fusarium verticillioides* [16]. *B. megaterium* can produce the fengycin family of polypeptides. Fengycin inhibits the growth of *F. moniliforme*, thus reducing fumonisin production [17]. *B. subtilis* can produce antifungal lipopeptides such as iturin, mycosubtilin, and fengycin. Fengycin attacks the integrity and structure of cell membranes of *Rhizopus stolonifer*, compromising cell membrane permeability and eventually leading to death [18, 19].

Although many studies have reported agents of *Bacillus* spp. having antagonistic activity as a biocontrol agent, very little has been reported about the antagonism of *B. methylotropicus* towards *M. fructicola*. The present study tested the antagonistic activity of *B. methylotrophicus* towards *M. fructicola* to determine the effectiveness of *B. methylotrophicus* against the growth of hyphae and spores of *M. fructicola* at the cellular level.

## 2 Materials and Methods

### 2.1 Development of the *M. fructicola*

In this study, we first isolated *M. fructicola* from infected peach fruits and then incubated the cultures on potato dextrose agar (PDA) at 28°C.

### 2.2 Isolation of bacteria from peach orchard soil

10 g soil samples were collected from five randomly selected locations within an area 20 cm from a peach tree trunk and at a depth of 30 cm in the soil. Then soil suspensions were prepared with a concentration of 10^-1^ g/mL. Then the soil suspensions underwent serial dilution yielding concentrations of 10^-2^ g/mL, 10^-3^ g/mL, 10^-4^ g/mL, 10^-5^ g/mL, and 10^-6^ g/mL in order to separate antagonist bacteria. Bacterial strains were separated by the dilution plate method: the diluent of 10^-3^ g/mL, 10^-4^ g/mL, and 10^-5^ g/mL was spread directly onto the surface of a peptone beef medium, then the bacterial strains were purified by the streaking plate method, and single colonies were inoculated onto the surface of the peptone beef medium and cultured at 37°C.

### 2.3 Screening trials

Antagonist bacterial strains were identified using the plate confrontation method. First, *M. fructicola* was inoculated in the middle of a PDA plate medium. Then the strains of bacteria isolated from the soil were inoculated at three equidistant positions, each 2.5 cm from the block of *M. fructicola*, and the plate was then incubated at 28°C for 7 days. Each treatment was repeated three times. Antagonistic effects were documented.

### 2.4 Characterization and identification of antagonistic bacteria

Using TIANamp Bacteria DNA kits (TIANGEN, China), all bacterial genomic DNA was extracted from bacterial strain XJ-C growing on the plate. PCR amplification and sequencing of the 16S rDNA gene were carried out in a 25 μl reaction mixture. The procedure was processed using the general forward primer 5′-AGAGTTTGATCCTGGCT

CAGAACGAACGCT-3′ and reverse primer 5′-AGAGTTTGATCCTGGCTGAG-3′ under the following conditions. Template DNA was denatured at 95°C for 5 min followed by 30 cycles at 94°C for 30 s. Annealing was performed at 58°C for 30 s and extension at 72°C for 15 min. The final step was carried out at 72°C for 10 min and then 4°C until infinity using a Touch Thermal Cycler (C1000, Bio-Rad, USA). A DNA template in sterile ddH_2_O, sequenced in Sangon Biotech (Beijing), was used as the negative control. The sequences were submitted to GenBank and were assigned accession numbers (MG752876). System development analysis of the clustering evolutionary tree was constructed by the neighbor joining method (n-j) in MEGA 6.0 software. Confidence limits of phylogenetic trees were estimated using bootstrap analysis (1,500 replications). Reference sequences were retrieved from GenBank under the accession numbers indicated in the tree.

Citrate utilization, starch hydrolysis, oxidative reactions, and more tests were carried out according to methods in the Common Bacterial System Identification Manual to determine the physiological and biochemical identification of the antagonistic bacteria [20].

### 2.5 Antagonistic activity of strain XJ-C against *M. fructicola in vitro*

Twelve fresh fruits, leaves, and shoots from peach trees were infested with *M. fructicola* according to the following procedure *in vitro*. All selected tissues were normal and at a similar developmental stage and harbored no plant diseases or insect pests. Prior to treatment, the bacterial strain XJ-C was concentrated to 1×10^7^ CFU/mL, 1×10^8^ CFU/mL, or 1×10^9^ CFU/mL. Sterile water was used as a control, and each treatment was repeated three times. One of each treatment was randomly selected to take photos.

For the fruit treatment, whole fruits were rinsed with sterile water three times then air-dried. We used a modified inoculation method as described by Li [21]. A sterile puncher was used to make a small hole about 5 mm deep and wide on the equator of each fruit. The fruits were then immersed in the different solutions of strain XJ-C suspension for 4 hours. Then a plug of *M. fructicola* was placed in the holes of the fruits before incubation in a controlled-environment chamber (28°C) for 3 days followed by observation of the extent of infection.

Leaves and shoots were surface-disinfected by immersing in 1% NaClO solution for 2 min and then rinsing with sterile water 3 times. Using a sterile scalpel, leaves at similar developmental stages were wounded at six spots at the mesophyll along the main vein. Wounds were approximately 1 mm in length. Each healthy shoot was wounded once with a sterile scalpel on the phloem. Wounded leaves and shoots were immersed in different solutions of XJ-C for 4 hours. Then a block of *M. fructicola* was placed in each wound in the leaves and shoots before incubation in a controlled-environment chamber (28°C) for 3 days followed by observation of the extent of infection.

*M. fructicola* infections of the fruit, leaves, and shoots were quantified as follows:

Incidence（%）= Lesion number of treated fruits / number of treated fruits× 100%

Inhibition rate (%) =[Lesion size of untreated fruits(cm^2^) - Lesion size of treated fruits(cm^2^)]/ Lesion size of control (cm^2^)× 100%

### 2.6 Microbiological analysis of the inhibition effect of *M. fructicola* by the strain XJ-C

Samples for optical, scanning electron, and transmission electron microscopy were taken from fresh *M. fructicola* agar diffusion plates. The samples were divided into two categories: (1) no inhibition observed in the growth of the fungi, as in the CK group, and (2) inhibition observed, apparently in the growth of the fungi, as in the experimental group.

For optical microscopy, samples were picked by a sterile inoculation hook, placed on microslides, coated with sterile water, and observed under the microscope (L135A).

Using a sterile blade, 3 × 3 mm samples of *M. fructicola* were cut from fungus cake and prepared for SEM. The preparation followed the methods of Rautio *et al*. [22] and Yang *et al*. [23]. Briefly, specimens were fixed with 2.5% glutaraldehyde followed by 1% osmium tetroxide at room temperature, dehydrated with ethanol, critical-point dried, coated with gold, and observed by SEM (TS-5136SB).

TEM samples were prepared using a method similar to that used for SEM. All excised samples were cut from fungus cake with a sterile blade into 2 × 3 mm sections. The specimens were pre-fixed in 2.5% glutaraldehyde for 5-6 h at room temperature, washed 5 times with 0.1 M phosphate buffer (PBS, pH 7.2), post-fixed with 1% osmium tetroxide for 1.5 h, washed 8 times with PBS, dehydrated with ethanol and acetone, embedded in spur resin, sectioned into 10 μm slices, dyed, and observed by TEM (H-7650) [24, 25].

### 2.7 Statistical analysis

Data collected were subjected to analysis of variance implemented in SPSS statistics 17.0. The means were separated using Duncan Multiple Range Test at the 0.05 level of significance.

## 3 Results

### 3.1 Screening trials

**Figure 1:**
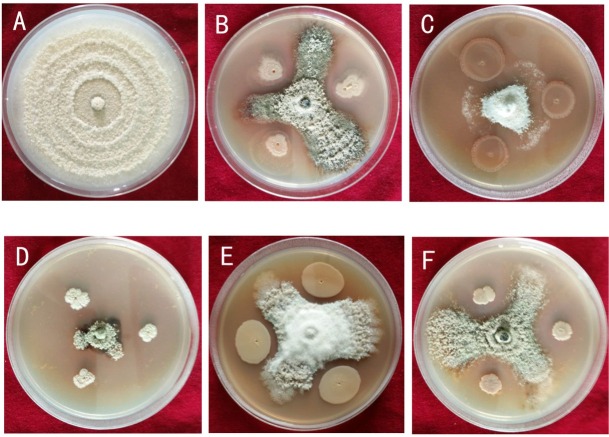
The antagonistic effects of XJ-A through E against *M. fructicola* (plate confrontation method) shows: Normal growth of *M. fructicola* (CK) (A); Growth of *M. fructicola* inhibited by XJ-A (B); Growth of *M. fructicola* inhibited by XJ-B (C); Growth of *M. fructicola* inhibited by XJ-C (D); Growth of *M. fructicola* inhibited by XJ-D (E); Growth of *M. fructicola* inhibited by XJ-E (F).

A total of 47 strains were originally isolated from the peach orchard soil, including 20 strains of bacteria. Based on results of the plate confrontation trial, 5 of those strains showed a wide range of antagonistic activity against *M. fructicola* (Fig 1). The inhibition rate of XJ-A to E was 73.68%, 76.31%, 81.57%, 60.52%, and 65.78%, respectively, and there were significant anti-fungal effects (Table1). The average radius of *M. fructicola* colonies inoculated with CK or XJ-A to E was 3.8 cm, 1 cm, 0.9 cm, 0.7 cm, 1.5 cm, and 1.3 cm, respectively. Notably, the strain XJ-C showed a significant effect on *M. fructicola* mycelial growth (Table 1, Fig 1), so it was selected for further characterization and investigation.

**Table 1:** The inhibition by each bacterium against *M. fructicola*.

Strain No.	Radius of the colony (cm)	Inhibition rate (%)
CK	3.80**±** 0.10 ^c^	**-**
XJ-A	1.00± 0.20 ^a^	73.68^a^
XJ-B	0.90± 0.10 ^b^	76.31^a^
XJ-C	0.70± 0.20^b^	81.57^b^
XJ-D	1.50±0.20^b^	60.52^c^
XJ-E	1.30±0.10^b^	65.78^a^

Means signed by the same letter differ not significantly according to duncann-test at P < 0.05.

### 3.2 Identification of strain XJ-C

**Figure 2:**
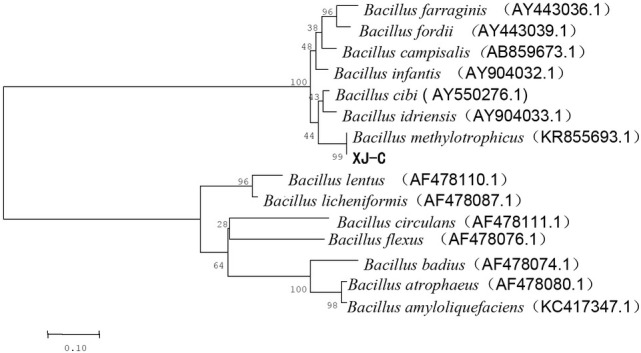
The scale bar at the bottom indicates genetic distance units based on Nei’s genetic distance.

The phylogenetic tree of 16S rDNA gene sequences revealed that strain XJ-C shared the greatest similarity with that of *B. methylotrophicus* (Fig 2). A comparison of the cultural and morphological characteristics of strain XJ-C was similar with those of *B. methylotrophicus*, including a creamy white appearance and folded surface. Table 2 shows that the physicochemical properties of strain XJ-C are similar to that of *B. methylotrophicus*. These results indicate that strain XJ-C is closely related to *B. methylotrophicus*.

**Table 2:** Biochemical analysis of strain XJ-C.

Project	XJ-C	*B. methylotophicus*	Project	XJ-C	*B.methylotophicus*
Acid is produced			Growth pH range		
Arabic sugar	+	+	pH=5 - pH=8	+	+
Mannitol	+	+	Citrate utilization	+	+
Xylose	+	+	Catalase	+	+
Glucose	+	+	Indole reaction	-	-
Temperature			Starch hydrolysis	+	+
4°C -20°C	-	-	Nitrate reduction	+	+
30°C-50°C	+	+	Phenylalanine deaminase	-	-
NaCl tolerance			Casein hydrolysis	+	+
1% -2%	+	+	Gram test	+	+
5 % -10 %	-	-			

Note: ‘+’positive reaction, ‘−’negative reaction.

### 3.3 The antagonist activity of strain XJ-C *in vitro*

**Figure 3:**
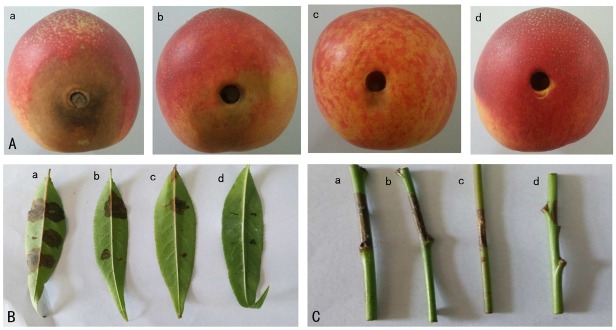
Antagonistic effects of strain XJ-C against *M. fructicola* in fruits, leaves, and shoots of the peach tree: Peach fruits (A); Peach leaves (B); Peach shoots (C). Sterile water as control (a); The concentration of XJ-C is 1×10^7^ CFU/mL (b); The concentration of XJ-C is 1×10^8^ CFU/mL (c); The concentration of XJ-C is 1×10^9^ CFU/mL (d).

The fruits, leaves, and shoots not treated with strain XJ-C (control) were infected by *M. fructicola* (Fig3A-a, 3B-a and 3C-a). Tissues treated with strain XJ-C at all concentrations (1×10^7^ CFU/mL, 1×10^8^ CFU/mL, and 1×10^9^ CFU/mL) effectively inhibited the growth of *M. fructicola* (Fig 3A-b-d, 3B-b-d and 3C-b-d). As the XJ-C concentration increased, the incidence of disease decreased, disease spot area was reduced, and the disease control effect increased for each tissue. The highest strain XJ-C concentration of 1×10^9^ CFU/mL displayed a significant anti-fungal effect, and significant anti-fungal differences was observed on the fruits, leaves, and shoots treated with strain XJ-C at all concentrations (Table 3).

**Table 3:** Inhibition of *M. fructicola* in peach fruits, leaves, and shoots treated with different concentrations of strain XJ-C.

Various tissue of peach trees	Concentration of bacterium (CFU/mL)	Incidence (%)	Radius of disease spot (cm)	Inhibition rate (%)
	a：CK	100^a^	4.40±1.56^d^	-
Peach fruit	b: 1×10^7^	66.7^a^	4.08±1.40^c^	7.27 ^c^
	c: 1×10^8^	66.7^a^	3.14±0.94^c^	28.61^c^
	d: 1×10^9^	33.3^a^	1.57±0.62^a^	64.31^a^
	a：CK	100^a^	1.13±0.3^a^	-
Leaves	b: 1×10^7^	61.1^a^	0.78±0.31^a^	30.95^a^
	c: 1×10^8^	38.9^a^	0.38±0.31^a^	66.36^b^
	d: 1×10^9^	22.2^a^	0.03±0.02^b^	97.34^b^
	a：CK	100^a^	1.40±0.06^a^	-
shoots	b: 1×10^7^	66.7^a^	1.20±0.04^a^	14.28^c^
	c: 1×10^8^	66.7^a^	1.00±0.04^a^	28.56^c^
	d: 1×10^9^	33.3^a^	0.50±0.02^a^	64.28^a^

Means signed by the same letter differ not significantly according to duncann-test at P < 0.05

### 3.4 Effects of strain XJ-C on the microstructure of *M. fructicola* under OM

**Figure 4:**
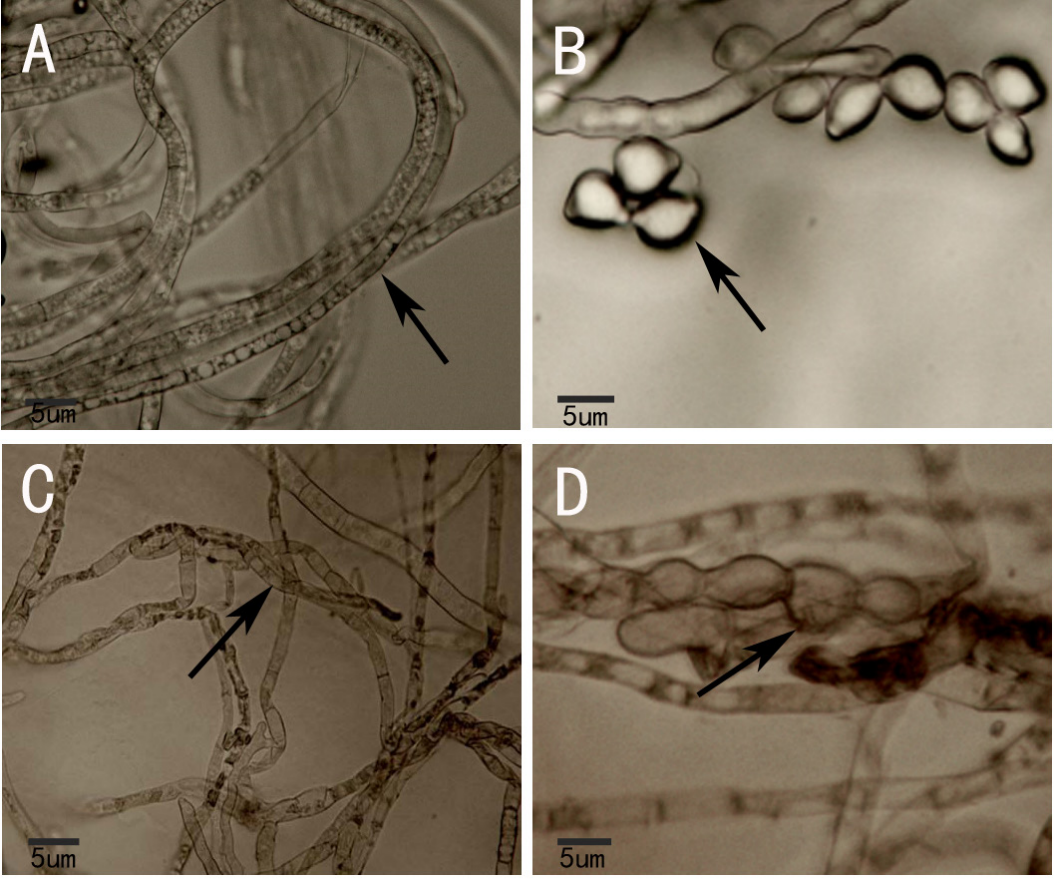
The effect of XJ-C on the structure of *M. fructicola* under OM: Normal mycelium of *M. fructicola* is full and smooth (A); Normal spores of *M. fructicola* are plump and lemon-shaped (B); The mycelium and spores of *M. fructicola* are shrunken under inhibition by strain XJ-C (C and D).

Under the optical microscope, normal mycelium of *M. fructicola* was full and smooth (Fig. 4A), and its spores were plump and lemon-shaped (Fig 4B). After inhibition by strain XJ-C, the mycelium and spores of *M. fructicola* were shrunken (Fig 4C and D).

### 3.5 Effects of strain XJ-C on the ultrastructure of *M. fructicola* under EM

**Figure 5:**
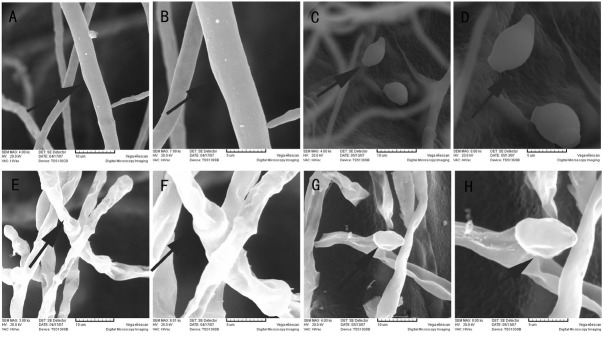
The effect of strain XJ-C on the structure of *M. fructicola* cells under SEM: Normal hyphae of *M. fructicola* are stretched and uniform, the surface is smooth and plump (A and B); Spores of *M. fructicola* are lemon-shaped with smooth surfaces (C and D); The shape and surface of affected hyphae are deformed (E and F); Affected spores are deformed with shrunken surfaces (G and H).

Under SEM, normal mycelium of *M. fructicola* was stretched and uniform with smooth, plump surfaces (Fig 5A and B). Treatment with strain XJ-C caused *M. fructicola* mycelium to grow abnormally. Mycelium shape and surfaces were deformed (Fig 5E and F). Normally, spores of *M. fructicola* appear lemon-shaped with smooth surfaces (Fig 5C and D), but their shape was deformed and their surfaces shrunken after treatment (Fig 5G and H).

**Figure 6:**
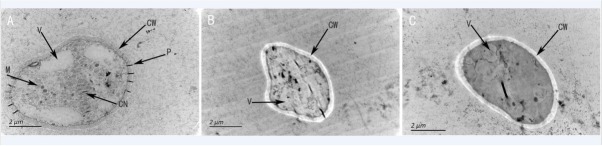
The effect of strain XJ-C on the structure of *M. fructicola* cells under TEM: An untreated, normal cell of *M. fructicola* clearly shows mitochondria, ribosomes, vacuoles, cell wall, and even plasmodesma (A); A cell of *M. fructicola* inhibited by strain XJ-C is damaged. The cell wall is transparent, the organelles have disappeared, and the intracellular vacuole is deformed (B and C). Mitochondria (M), cell nucleus (CN), vacuoles (V), cell wall (CW), plasmodesma (P).

Under the TEM, the mitochondria, ribosomes, vacuoles, cell wall, and even the plasmodesma were clearly visible in a normal cell of *M. fructicola* (Fig 6A). However, cells of *M. fructicola* showed obvious changes and damage after treatment with strain XJ-C. The cell wall was transparent, the organelles had disappeared, and the intracellular vacuoles were deformed (Fig 6B and C).

## 4 Discussion

In recent years, more and more people have become engaged in the research of biological control, which has become an effective approach to plant disease management because of its minimal toxicity to humans and other non-target species [26, 27]. *Bacillus sp*. is abundant in the rhizosphere and commonly present on the soil surface [28]. Most strains of *Bacillus sp*. that are antagonistic towards fungi have been isolated from soil. Research has confirmed the important role that *B. amylobacillus* and *B. subtilis* play as *M. fructicola* antagonists. For example, *B. subtilis* antagonizes *M. fructicola* by producing a wide variety of antimicrobial compounds including fengycin, iturin, and surfactin [18, 29]. The agent bacillus B91 can significantly reduce the formation of spores [30]. The agent *B. amyloliquefaciens* C06 produced two antimicrobial compounds belonging to the iturin and fengycin families, and those two antimicrobial compounds combine to inhibit the conidial germination of *M. fructicola* [31]. Although there are many reports of *Bacillus spp*. inhibiting the growth of *M. fructicola*, there are few reports on the inhibition of *M. fructicola* by *B. methylotrophicus*. In our study, one strain of bacillus that we call XJ-C was isolated from the soil of a peach orchard. The bacterium was identified by molecular and physicochemical methods as *B. methylotrophicus*, and based on the results of experiments on peach fruit, leaves, and shoots *in vitro*, it was found to have an antagonistic effect on *M. fructicola*. After treatment with XJ-C, the mycelium and spores of *M. fructicola* were damaged, and cell structure was destroyed. Previous studies have found similar results in this field. BmB 1 metabolites of *B. amyloliquefaciens* were able to disrupt the cell walls of *Rhizoctonia sp*., *Sclerotium sp., and Pythium sp*., resulting in the discharge of cellular contents [32]. Hyphal morphology of *Magnaporthe grisea* became irregular when that fungus was treated with specific biological control agents produced by bacteria [33].

We assert that more attention should be focused on the substantial inhibitory effect of *B. methylotrophicus* on plant pathogenic fungi. Jemil *et al*. found that the lipid-peptides separated from *B. methylotrophicus* could effectively antagonize fungal diseases by destroying cell membrane integrity [32]. *B. methylotrophicus* isolated from the stem tissue of *Bacopa monnieri* contained the genes for surfactin, iturin, and type I polyketide synthase (PKS), all of which directly inhibit pathogen growth by producing surfactin and iturin [12]. Shrestha *et al*. [34] found that *B. methylotrophicus* isolated from rice seedlings can effectively inhibit fungal germination. The active substance phenaminomethylacetic acid was extracted by TLC and HPLC from the fermentation liquid of *B. methylotrophicus*, and its inhibitory efficiency was strong when used against *M. oryzae* [35]. The genes that encode the biosynthetic enzymes mersacidin and amylolysin, which have antibacterial activity, were found in the genome of *B. methylotrophicus* [36]. Lipopeptides - surfactins, iturins and fengycins - were detected in *B. methylotrophicus* by mass spectrometry analysis, and these are the main compounds that confer biocontrol ability [37]. At present, the antibacterial substances that were found and applied to plant disease control include lipid peptides, peptides, phospholipids, polyene, amino acids, nucleic acids, etc. [38, 39, 40]. Based on the results of antibacterial activity of *B. methylotrophicus* in this experiment and the separation of active substances carried out by our group, our results showed that the antagonistic activity of n-butanol extract was the best, though the specific active substance was not determined (unpublished). In subsequent studies, we plan to explore bacillus antagonism mechanisms, first investigating compounds that will play an important role against *M. fructicola*. In previous experiments, research showed that *Bacillus spp*. can inhibit the growth of pathogenic fungi by releasing one or several antimicrobial compounds such as antibiotics and antimicrobial peptides. Undoubtedly, with continued research into antimicrobial compounds, more and more such compounds will be extracted from antifungal strains for applications in agricultural production. This study has provided an experimental foundation and theoretical support for field experiments as well as necessary information to advance the use of *Bacillus spp*. for biological control of plant disease.
